# Zn(II) and Co(II) 3D Coordination Polymers Based on 2-Iodoterephtalic Acid and 1,2-bis(4-pyridyl)ethane: Structures and Sorption Properties

**DOI:** 10.3390/molecules27041305

**Published:** 2022-02-15

**Authors:** Alexander S. Zaguzin, Taisiya S. Sukhikh, Ilyas F. Sakhapov, Vladimir P. Fedin, Maxim N. Sokolov, Sergey A. Adonin

**Affiliations:** 1 South Ural State University, Lenina St. 76, 454080 Chelyabinsk, Russia; zaguzin@niic.nsc.ru (A.S.Z.); sakhapovilyas@mail.ru (I.F.S.); 2 Nikolaev Institute of Inorganic Chemistry, SB RAS, Lavrentieva St. 3, 630090 Novosibirsk, Russia; sukhikh@niic.nsc.ru (T.S.S.); cluster@niic.nsc.ru (V.P.F.); caesar@niic.nsc.ru (M.N.S.); 3 Arbuzov Institute of Organic and Physical Chemistry, RAS, Arbuzov St. 8, 420088 Kazan, Russia

**Keywords:** zinc, metal-organic frameworks, coordination polymers, carboxylates, sorption

## Abstract

Metal-organic frameworks [M_2_(2-I-bdc)_2_bpe] (M = Zn(II) (**1**), Co(II) (**2**), 2-I-bdc = 2-iodoterephtalic acid, and bpe = 1,2-bis(4-pyridyl)ethane) were prepared and characterized by X-ray diffractometry. Both compounds retain their 3D structure after the removal of guest DMF molecules. Selectivity of sorption of different organic substrates from the gas phase was investigated for both complexes.

## 1. Introduction

Metal-organic frameworks, or MOFs, constitute a very important class of coordination compounds that remain a “hot topic” within the last decades [1,2,3,4,5,6,7,8,9]. Among the potential application areas making MOFs attractive objects, there are sensors, including detection of explosives, toxins, or even cancer biomarkers [8,10,11,12,13,14,15,16], catalysis [17,18,19,20,21,22,23], and separation of different substrates (hydrocarbons [24,25], etc.). The latter field is especially important since different MOFs can demonstrate remarkable sorption selectivity towards mixtures that are relevant to those occurring in real industrial processes–for example, cyclohexane/benzene [26,27,28,29,30,31], hexane/cyclohexane, [32] acetylene/alkanes [33,34,35], etc. MOFs can also be used for the removal of contaminants (both organic and inorganic) from water [36,37,38].

The sorption ability of MOFs is closely related to the features of supramolecular contacts between organic ligands and guest molecules. These non-covalent interactions can be different. Usually, the main role is played by hydrogen bonding, but reports also appear where other types of supramolecular interactions, in particular, halogen bonding (XB) [39], have a major contribution. These examples are yet rare [40,41,42], but, in our opinion, they demonstrate that this topic can evolve further.

Among the great variety of building blocks able to form XB, there are halogenated hydrocarbons (alkanes [43,44,45,46] and, less commonly, alkynes [47,48]), perfluorinated iodoarenes [49,50,51,52], di- and polyhalogens [53,54,55], complexes with halogen-substituted pyridines [56,57,58,59,60], etc. For the design of MOFs, halogen-substituted aromatic polycarboxylic acids seem to be the suitable option: it can be assumed that halogens, especially iodine, can feature the presence of sigma-hole due to the impact of electron-withdrawing carboxylic groups. Of this family, 2-iodoterephtalic acid (2-I-bdc) is easily available [61]; very recently, we reported in [62] corresponding on [Zn_2_(I-bdc)_2_dabco] MOF and compared its sorption selectivity towards different organic substrates with parent [Zn_2_bdc_2_dabco]. Continuing this work, we changed the N,N-linker ligand for 1,2-bis(4-pyridyl)ethane (bpe) and hereby present two new coordination polymers-[M_2_(2-I-bdc)_2_bpe] (M = Zn(II) (**1**) and Co(II) (**2**). Both compounds feature 3D structure; sorption selectivity was investigated and compared for **1** and **2.**

## 2. Results and Discussion

**Figure 1:**
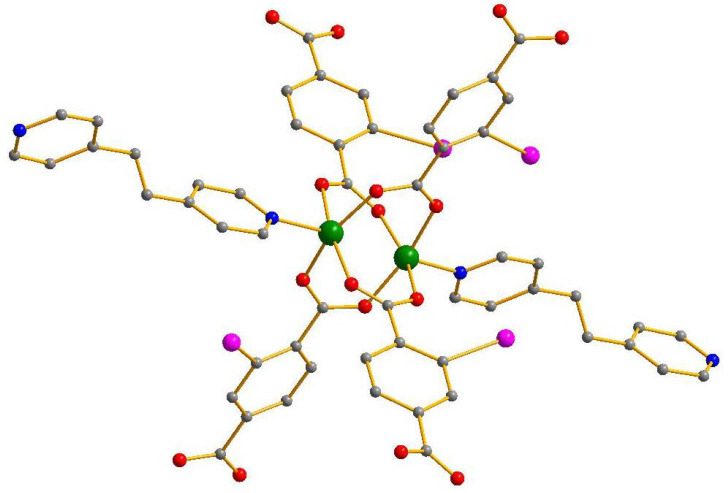
Coordination unit in structures **1** and **2**. Metal green, C grey, O red, I purple, N deep blue. H atoms are omitted for clarity, only one of three possible positions of I is shown.

The structures of **1** and **2** are based on similar building blocks. In both cases, there form dimeric paddlewheel-type {M_2_(OOCR)_4_(bpe)_2_} units (Figure 1), very common for carboxylate complexes. The M-O bond lengths in **1** and **2** are 2.029–2.051 and 2.013–2.036 Å, respectively, the M-N bonds are 2.019–2.030 and 2.044 Å, respectively. The iodine atoms of 2-I-bdc ligands are disordered over three positions in both structures (the occupancies are 0.444:0.307:0.249 and 0.641:0.160:0.199, respectively). In structure 1, one of two arene rings in bpe ligand is disordered as well (two positions with 0.68:0.32 occupancy).

**Figure 2:**
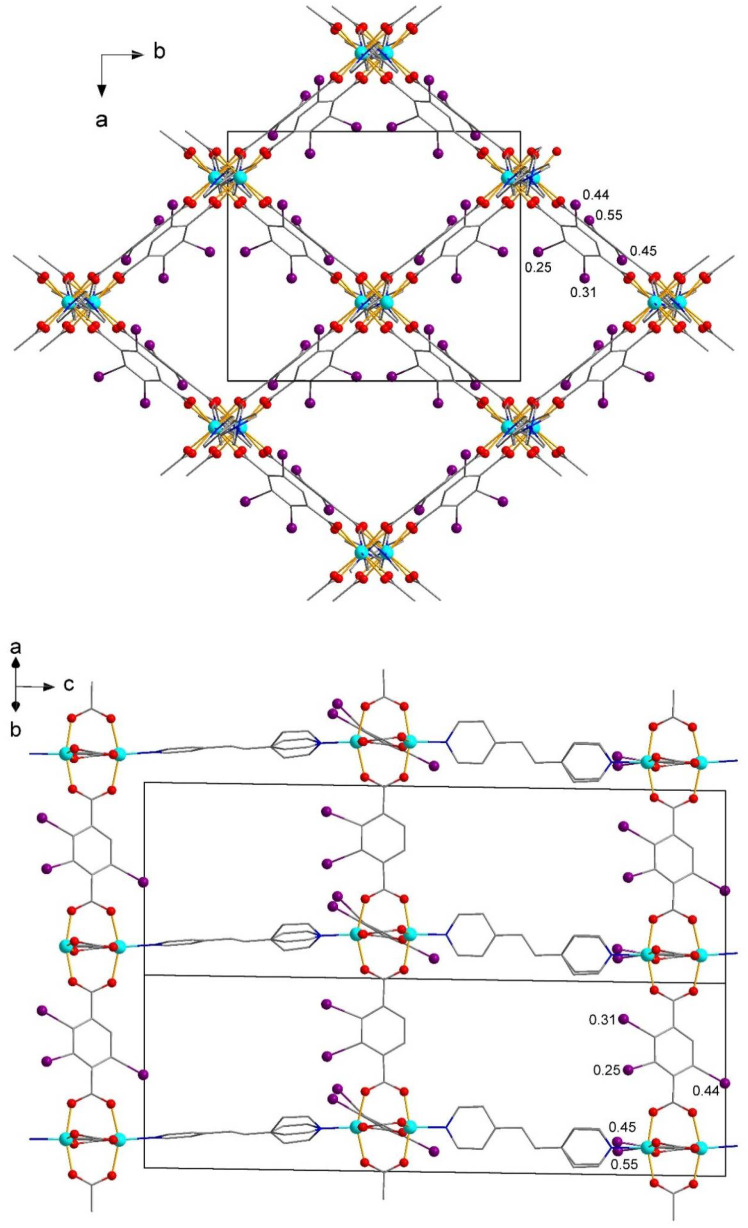
A fragment of the framework of **2**. Hydrogen atoms are omitted, the occupancy of disordered I atoms is shown.

The {M_2_(I-bdc)_4_} secondary building units are further connected via bpe ligands to a cuboid framework with rhombic-rod pores. Previously reported non-iodinated congener, [Zn_2_(bdc)_2_(bpe)] [63], reveals a tetragonal structure with bpe disordered over eight positions due to its proximity to a special position (4/mmm). Compound **1** comprises more ordered bpe (two crystallographically independent ligands) and two independent I-bdc ligands (Figure 2), thereby exhibiting a less symmetrical structure of ca. 4-fold increased cell volume as compared to [Zn_2_(bdc)_2_(bpe)]. For **1**, the non-equivalence of the bpe and I-bdc ligands manifest in a different relative arrangement of the aromatic rings and (in the case of I-bdc) in a different disorder pattern of I atoms.

**Figure 3:**
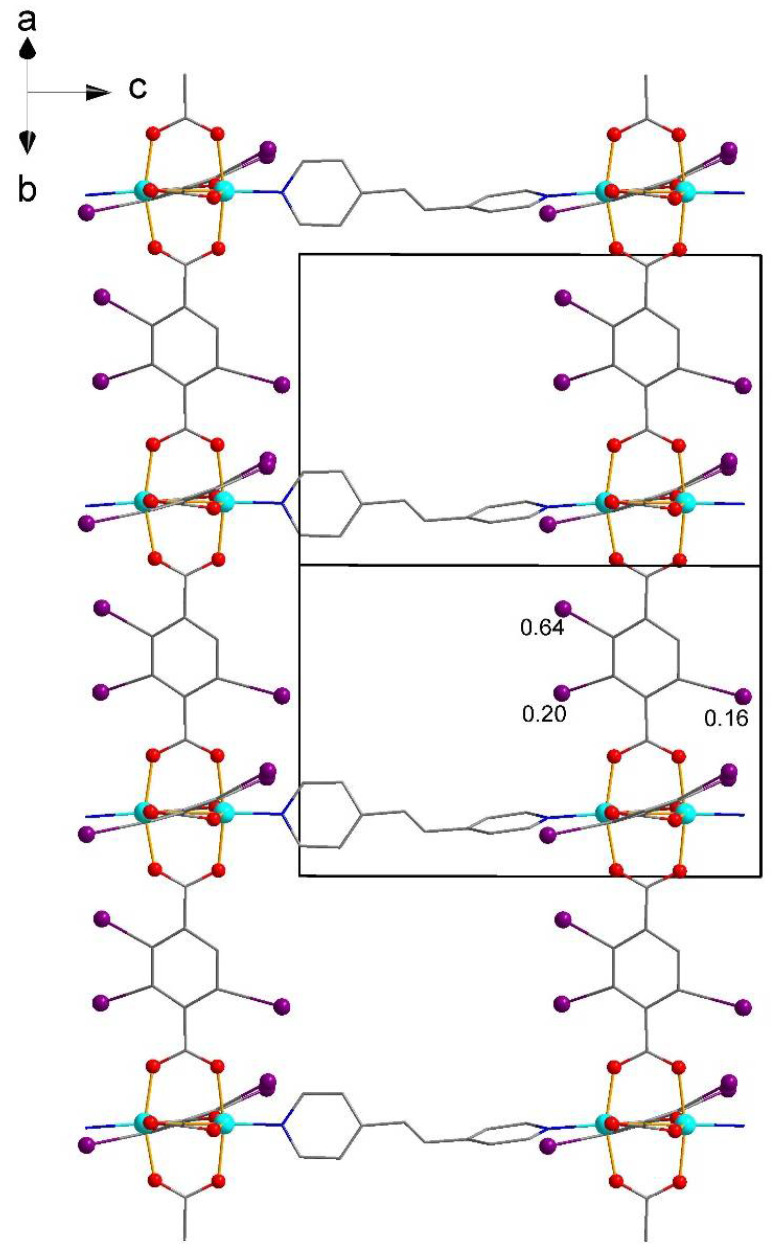
A fragment of the framework of **1**. Hydrogen atoms are omitted, the occupancy of disordered I atoms is shown.

**Figure 4:**
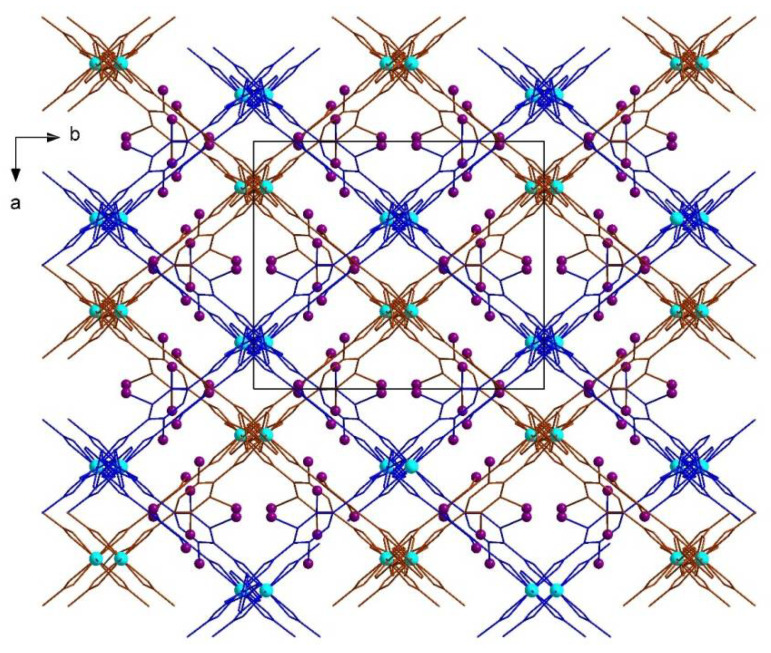
A fragment of the structures of the compounds on the example of **2** showing interpenetrated frameworks colored with blue and brown.

**Figure 5:**
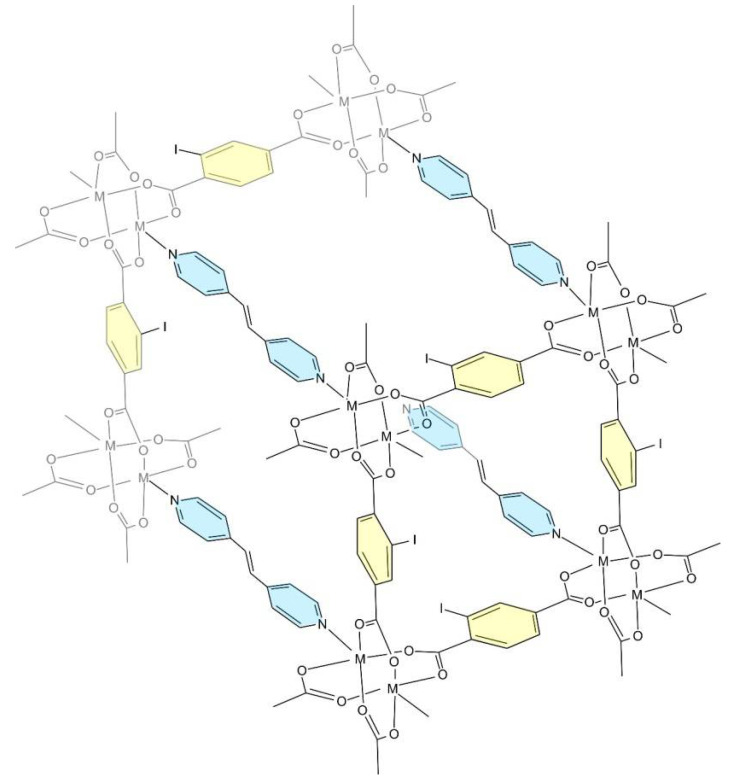
A fragment of the structures of the compounds in the example of **2** showing interpenetrated frameworks colored with blue and brown.

The structure of **2** is more symmetrical (namely, it has fewer translational symmetry elements per the same fragment of the structure), comprising only one independent I-bdc and half of bpe ligand (Figure 3). In structures of **1** and **2**, the frameworks show two-fold interpenetration (Figure 4), which reduces the volume of voids accessible for the inclusion of solvate molecules. The potential volume is estimated to be 22% and 25% from the total volume of structures **1** and **2**, respectively, although the actual values are higher due to the unaccounted volume taken by the disordered atoms. The topology of MOFs **1** and **2** is shown in Figure 5.

The experimental PXRD pattern for **1** resembles that for the sub-structure akin to **2** (Figure S1); compound **1** retains its structure after the removal of guest DMF molecules (see Experimental for details). The notable difference between the experimental and simulated patterns of **1** may arise from a slight change of the structure upon partial loss of solvate molecules under sample preparation and/or due to difference in the temperature of the single-crystal (150 K) and powder (298 K) experiments. To clarify this, we carried out PXRD experiments at 150 K for an as-synthesized sample under a small amount of the mother liquor; the data was in better agreement with the simulated pattern of the sub-structure. A few superstructural reflections were observed; however, they did not correspond to super-structure **1**. The presence of sub- and super-structures for the family of MOFs seemed to be common. For instance, related MOFs formulated as [Zn_2_(bdc)_2_(dabco)] (dabco is 1,8-diazabicyclooctane) revealed a variety of cuboid structures of the same topology that differ from each other mainly by spatial geometry of {Zn(bdc)} layers [62]. Relatively poor quality of the PXRD pattern of **1** did not allow us to evaluate the superstructural motif. The PXRD for a sample of **1** dried naturally, measured at 150 K (Figure S3, SI), slightly differed from that with the mother liquor, implying minor structural transformations upon partial loss of the solvate. It became similar to the room-temperature patterns for as-synthesized and activated samples (Figure S1). The same trend was observed for the PXRD patterns of sample **2** (Figures S2 and S4). Upon activation of sample **2**, the structure changes more noticeably, which was reflected in the appearance of strong superstructural reflections.

Sorption selectivity of **1** and **2** towards different organic substrates was examined using NMR (see Supplementary Information for NMR spectra). Results are given in Table 1.

Both **1** and **2** more readily absorbed 1,2-dichloroethane from its mixture with benzene than MOFs of the dabco family. Although the difference was not large, these results are rather inspiring. The best selectivity was demonstrated by **1** for benzene/cyclohexane mixtures—it exceeded one of [Zn_2_(bdc)_2_dabco]. Interestingly, the selectivity in 1-butanol/1-bromobutane pair was reversed for bpe and dabco series; the reasons for this effect are unclear.

## 3. Materials and Methods

All reagents were obtained from commercial sources and used as purchased. Solvents were purified according to the standard procedures. 2-iodoterephtalic acid was prepared according to the method reported earlier [61] and identified by its ^1^H and ^13^C NMR spectra.

**Synthesis of 1**

Seventy-four and a half micrograms (0.25 mmol) of Zn(NO_3_)_2_·6H_2_O, 73 mg (0.25 mmol) of 2-I-bdc, 23 mg (0.125 mmol) of bpe, and 8 mL of anhydrous DMF were placed into a glass ampoule which was sealed, kept in an ultrasonic bath for 10 min and kept at 120 °C for 48 h with slow cooling to room temperature—forming colorless crystals of **1** with a 79% yield.

**Synthesis of 2**

The procedure was the same as for **1**, using 119 mg (0.5 mmol) of CoCl_2_·6H_2_O, 146 mg (0.5 mmol) of 2-I-bdc, 46 mg (0.25 mmol) of bpe, and 7.5 mL of anhydrous DMF—forming deep blue colorless crystals of **2** with a 73% yield.

**Sorption of organic substrates**

The method was identical to one described by us earlier [62]. Prior to sorption experiments, samples of 1 or 2 were kept in excess of acetone for 48 h, followed by drying *in vacuo* (4 h, 60 °C) in order to eliminate guest DMF molecules. After that, the sample of MOF (50 mg) was placed into an open smaller vial, which was then placed into the closed bigger vial containing a mixture of organic substrates in 1:1 M ratio so that the liquid phase level is below the edges of the smaller vial. By this, the MOF sample was allowed to absorb organic substrates from the gas phase. After 48 h, the MOF sample was placed into a DMF:d^6^-DMSO mixture (1:1) and kept for 48 h again in order to desorb the substrates from the pores. The organic solvents and the ratio between the extracted organic substrates were established by comparison of relevant ^1^H NMR intensities (see SI).

**X-ray Diffractometry**

Data sets for single crystals of **1** and **2** were obtained at 150 K on a Bruker D8 Venture diffractometer (Bruker, Billerica, MA, USA) with a CMOS PHOTON III detector and IµS 3.0 source (mirror optics, λ(MoKα) = 0.71073 Å). Absorption corrections were applied with the use of the SADABS program. The crystal structures were solved using the SHELXT [64] and were refined using SHELXL [65] programs with OLEX2 GUI [66]. Atomic displacement parameters for non-hydrogen atoms were refined anisotropically. The available volume for solvate molecules is estimated using OLEX2 Solvent Mask Procedure to be minimum 214 Å3 and 240 Å3 per [M_2_(I-bdc)_2_(bpe)] formula unit for **1** and **2**, respectively; actual space is larger due to the disorder of iodine atoms. Details of XRD experiments are given in SI (Table S1). CCDC 2142027–2142028 contains the supplementary crystallographic data for this paper.

**Powder X-ray Diffractometry** (**PXRD**)

Powder XRD data for the compounds were collected at 150 K by a Bruker D8 Venture diffractometer (Bruker, Billerica, MA, USA) with a CMOS PHOTON III detector and ImS 3.0 microfocus source (CuK_α_ radiation, collimating Montel mirrors). Powder samples were mounted on a nylon loop with a small amount of epoxy resin [67,68]. By φ-scanning (360°), Debye diffraction patterns with continuous diffraction arcs were measured. To diminish the effect of the preferred orientations, five scans were made at different positions of a goniometer for ω from –240° to 0°. The external standard (Si) correction and integration were performed using the Dioptas program [69]. At 298 K, PXRD analysis was performed on Shimadzu XRD-7000 diffractometer (Shimadzu Scientific Instruments Incorporated, Kyoto, Japan) (CuK-alpha radiation, Ni–filter, linear One Sight detector, 5–50° 2θ range, 0.0143° 2θ step, 2 s per step).

**NMR Spectroscopy**

All NMR experiments were performed on a Bruker Avance III 500 MHz spectrometer (Bruker, Billerica, MA, USA) at room temperature (25 °C).

## 4. Conclusions

We prepared two new metal-organic frameworks based on M(II), 2-iodoterephtalic acid, and bpe linkers. Both complexes reveal interpenetrating 3D structures. The Zn-based complex **1** features a relatively high gas-phase sorption selectivity for benzene:cyclohexane mixture. Despite the overall structural similarity, their adsorption selectivity towards various organic substrates is different, demonstrating that even minor changes in structure can strongly affect these characteristics. Experiments aiming preparation of other M(II)-based MOFs with the same ligand combination, as well as with other iodine-substituted carboxylates, are underway in our group.

## Figures and Tables

**Table 1:** Selectivity of vapor adsorption by **1**, **2,** [Zn_2_(2-I-bdc)_2_dabco] and [Zn_2_(bdc)_2_dabco] [62] from equimolar mixtures of different organic substrates.

No.	Substrates	1	2	[Zn_2_(2-I-bdc)_2_dabco]	[Zn_2_(bdc)_2_dabco]
1	1,2-dichloroethane:benzene	2.54:1	2.57:1	1.5:1	1.2:1
2	Chloroform:benzene	3.3:1	1:1	1.3:1	2.0:1
3	Benzene:cyclohexane	43.8:1	10:1	15:1	25:1
4	1,2-dibromoethane:benzene	8.3:1	3.1:1	n/a	n/a
5	1-butanol:1-bromobutane	1.3:1	1.17:1	0.38:1	0.5:1

## Data Availability

Not applicable.

## Supplementary Materials

The following are available online: XRD, PXRD, and NMR data.

## Sample Availability

Samples of the compounds are not available from the authors.
