# Supplementary material for: Zn(II) and Co(II) 3D Coordination Polymers Based on 2-Iodoterephtalic Acid and 1,2-bis(4-pyridyl)ethane: Structures and Sorption Properties
Source: Molecules. 2022 Feb 15;27(4):1305. doi: 10.3390/molecules27041305 (PMC8876937; doi:10.3390/molecules27041305)
Supplement: Supplementary file 1 [file molecules-27-01305-s001.zip › molecules-1577127-supplementary.pdf]

Zn(II) and Co(II) 3D coordination polymers based on 2-iodoterephtalic acid and 1,2-bis(4-pyridyl)ethane: structures and sorption properties

Alexander S. Zaguzin, Taisiya S. Sukhikh, Ilyas S. Sakhapov, Vladimir P. Fedin, Maxim N. Sokolov and Sergey A. Adonin

Table S1. Crystal data and structure refinement for **1** and **2**

Identification code	**1**	**2**
Empirical formula	$C_{31}H_{23}I_2N_3O_9Zn_2$	$C_{34}Co_2H_{30}I_2N_4O_{10}$
Formula weight	966.06	1026.28
Temperature/K	150(2)	150(2)
Space group	*C*2/*c*	*C*2/*c*
a/Å	14.1684(4)	16.3143(14)
b/Å	16.5994(4)	14.5781(15)
c/Å	32.5179(8)	16.1189(16)
α/°	90	90
β/°	91.1000(10)	90.170(4)
γ/°	90	90
Volume/Å^3	7646.4(3)	3833.6(6)
Z	8	4
ρ_{calc}g/cm^3	1.678	1.778
μ/mm^{-1}	2.919	2.535
F(000)	3744.0	2008.0
Crystal size/mm^3	0.2 × 0.15 × 0.12	0.12 × 0.08 × 0.05
Radiation	MoKα (λ = 0.71073)	MoKα (λ = 0.71073)
2Θ range for data collection/°	3.964 to 54.22	2.526 to 51.358
Index ranges	-18 ≤ h ≤ 18, -20 ≤ k ≤ 21, -41 ≤ l ≤ 41	-18 ≤ h ≤ 19, -17 ≤ k ≤ 17, -19 ≤ l ≤ 19
Reflections collected	52403	9109
Independent reflections	8376 [R_{int} = 0.0599, R_{sigma} = 0.0369]	3593 [R_{int} = 0.0415, R_{sigma} = 0.0502]
Data/restraints/parameters	8376/127/447	3593/52/207
Goodness-of-fit on F^2	1.046	1.046
Final R indexes [I>=2σ (I)]	R_1 = 0.0818, wR_2 = 0.1967	R_1 = 0.0883, wR_2 = 0.2462
Final R indexes [all data]	R_1 = 0.0935, wR_2 = 0.2051	R_1 = 0.1016, wR_2 = 0.2632
Largest diff. peak/hole / e Å^{-3}	2.75/-2.65	2.12/-1.29

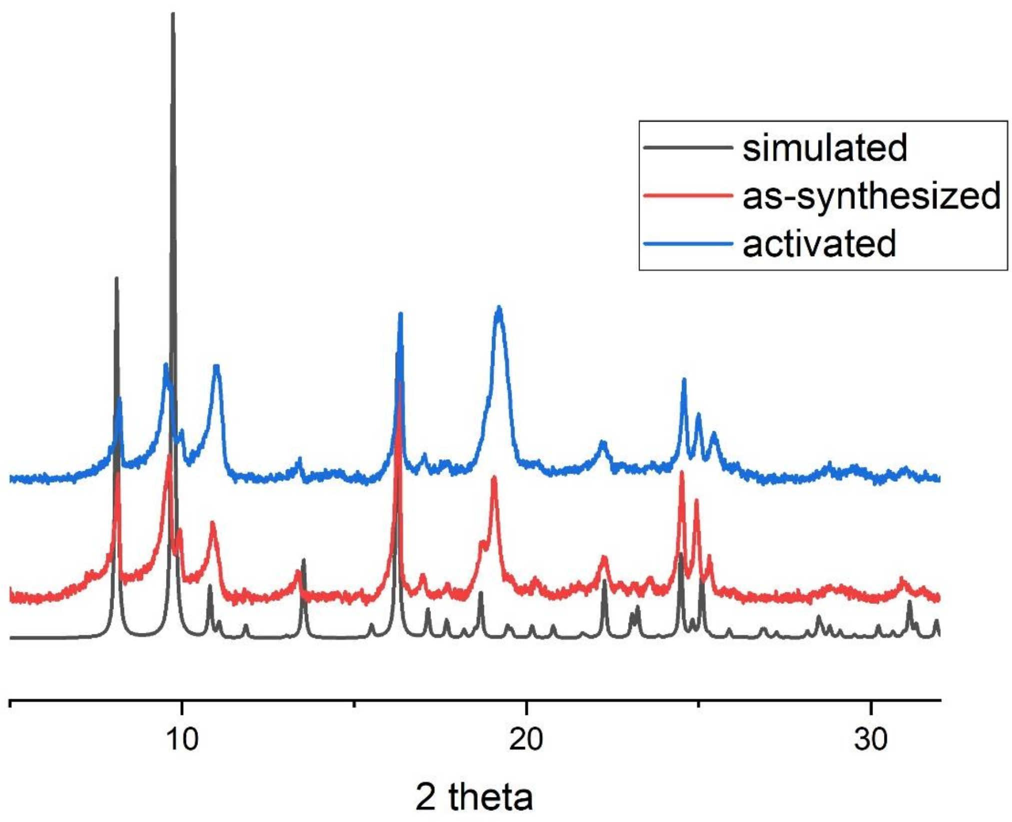

Figure S1. PXRD data for **1**

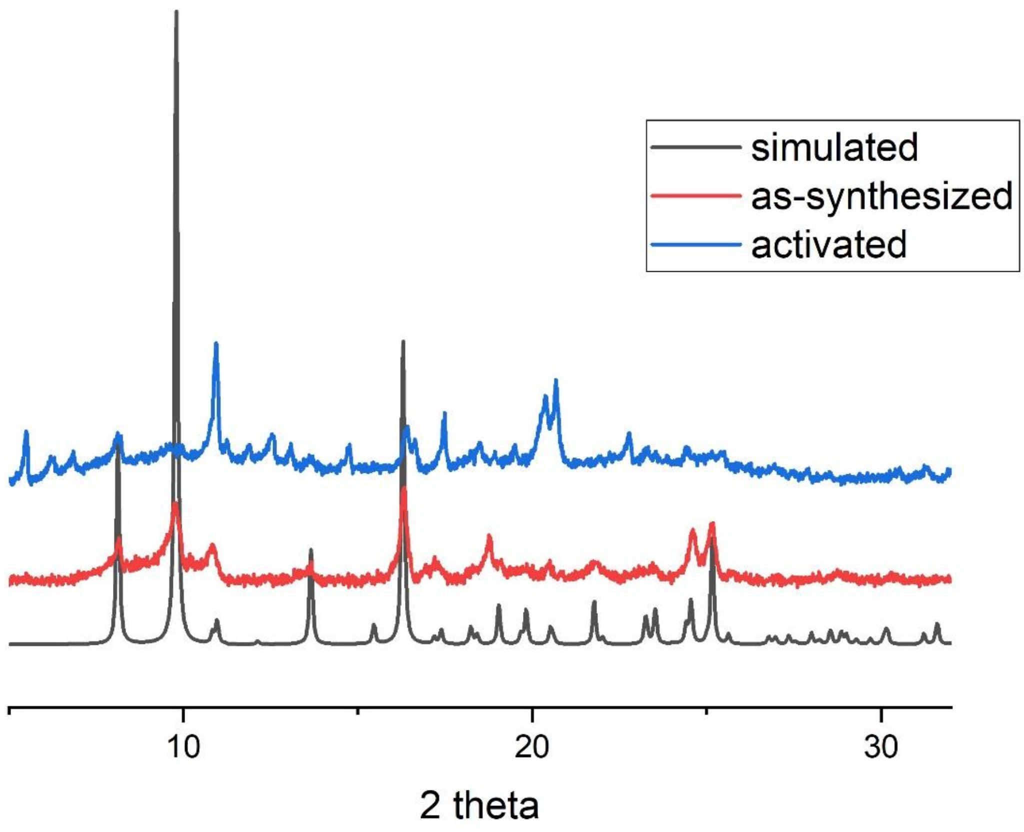

Figure S2. PXRD data for **2**

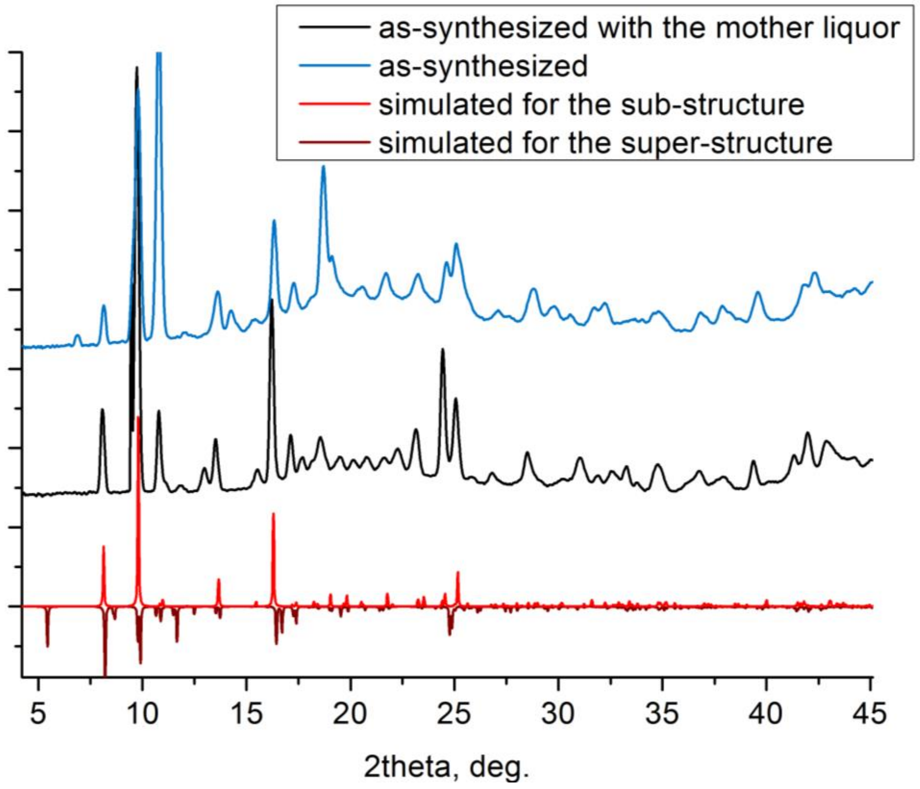

Figure S3. PXRD data for 1 at 150 K

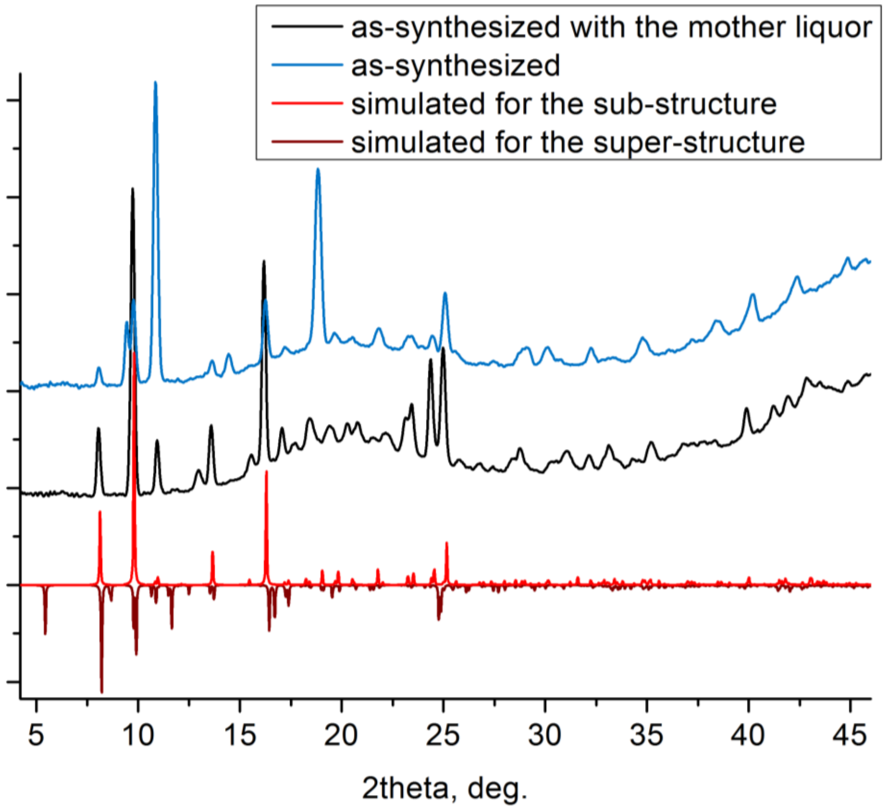

Figure S4. PXRD data for 2 at 150 K.

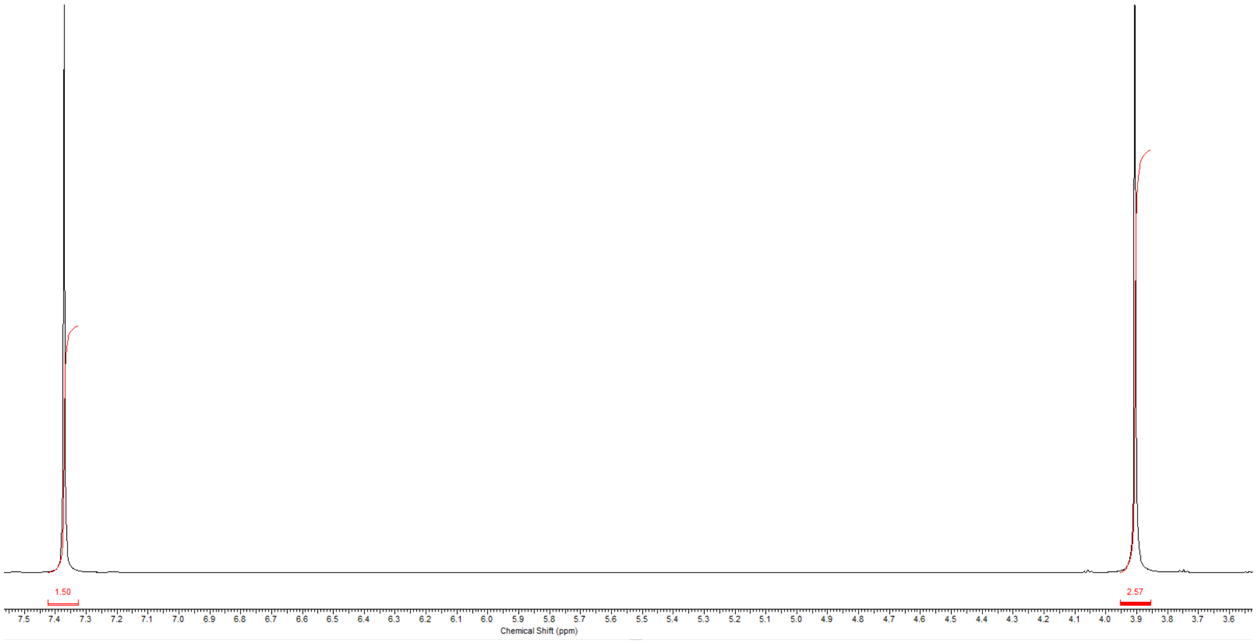

Figure S5. ^1H NMR spectrum, sorption experiment with **2**, benzene:dichloroethane

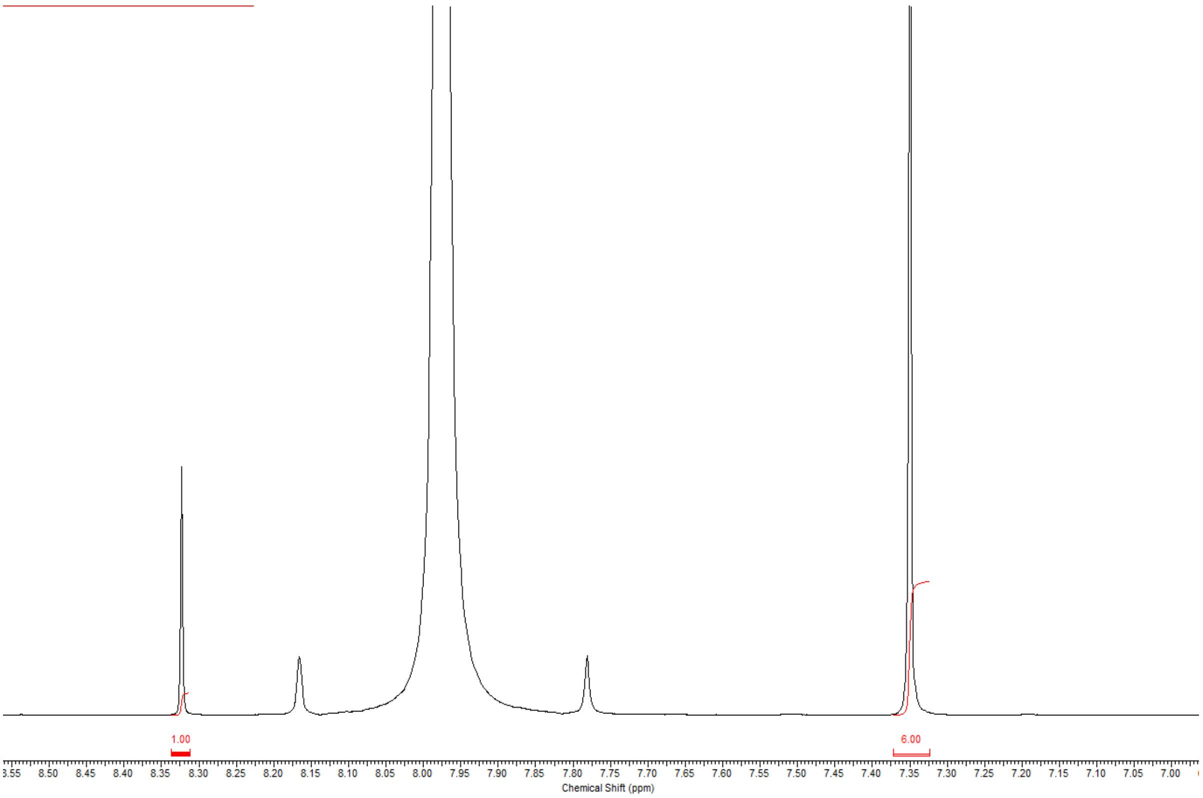

Figure S6. ^1H NMR spectrum, sorption experiment with **2**, benzene:chloroform

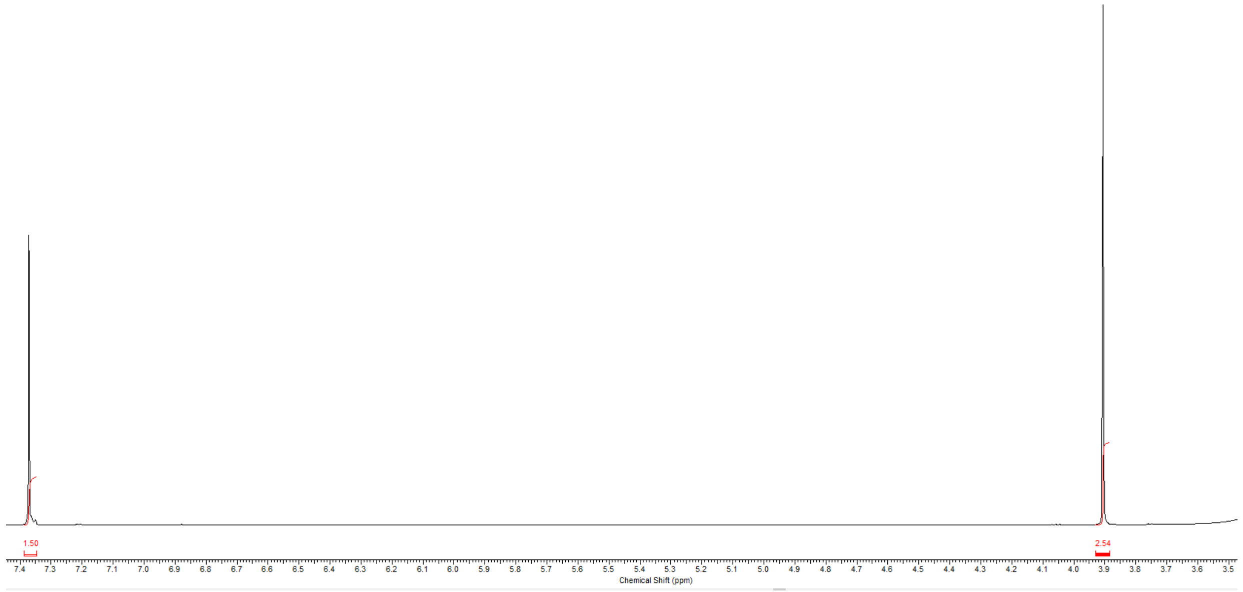

Figure S7. ^{1}H NMR spectrum, sorption experiment with **1**, benzene: dichloroethane

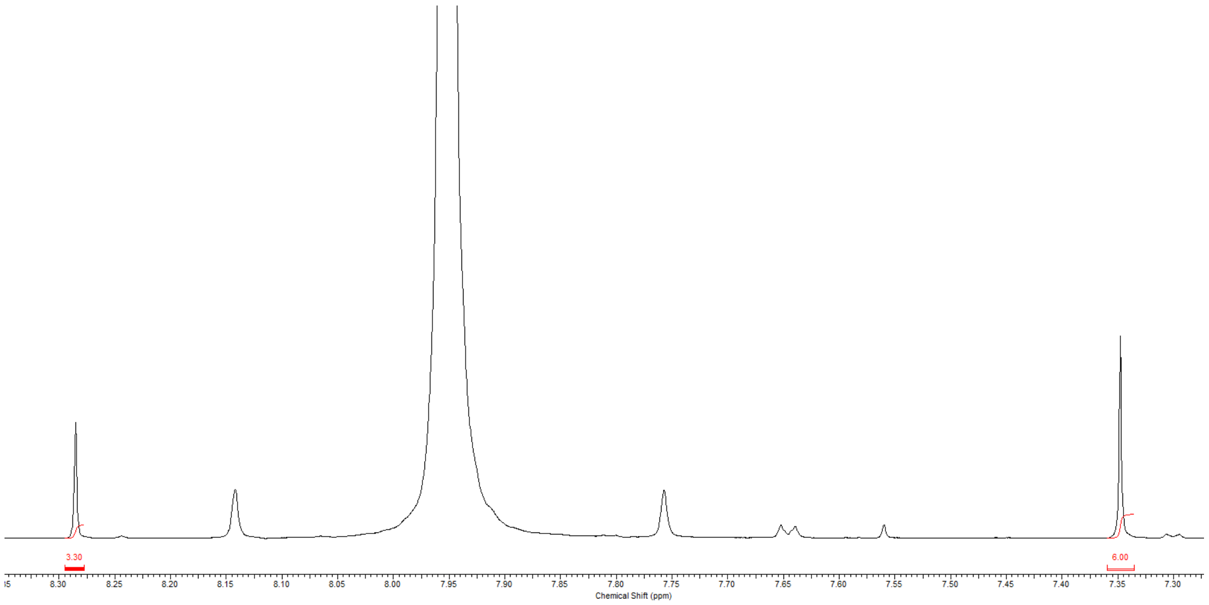

Figure S8. ^{1}H NMR spectrum, sorption experiment with **1**, benzene: chloroform

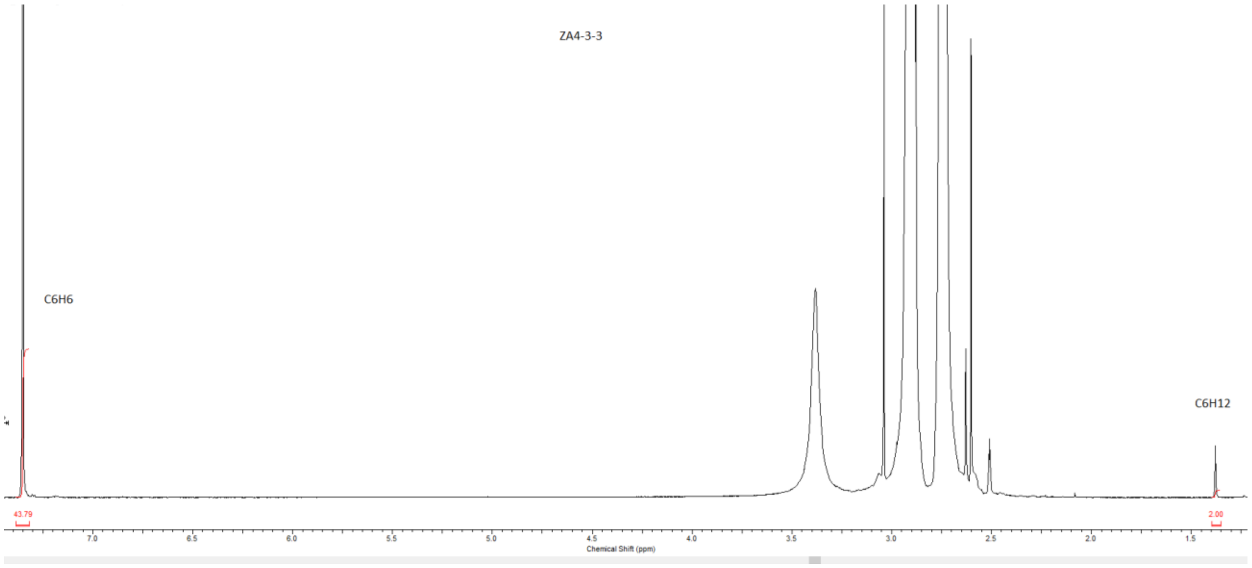

Figure S9. ^{1}H NMR spectrum, sorption experiment with **1**, benzene:cyclohexane

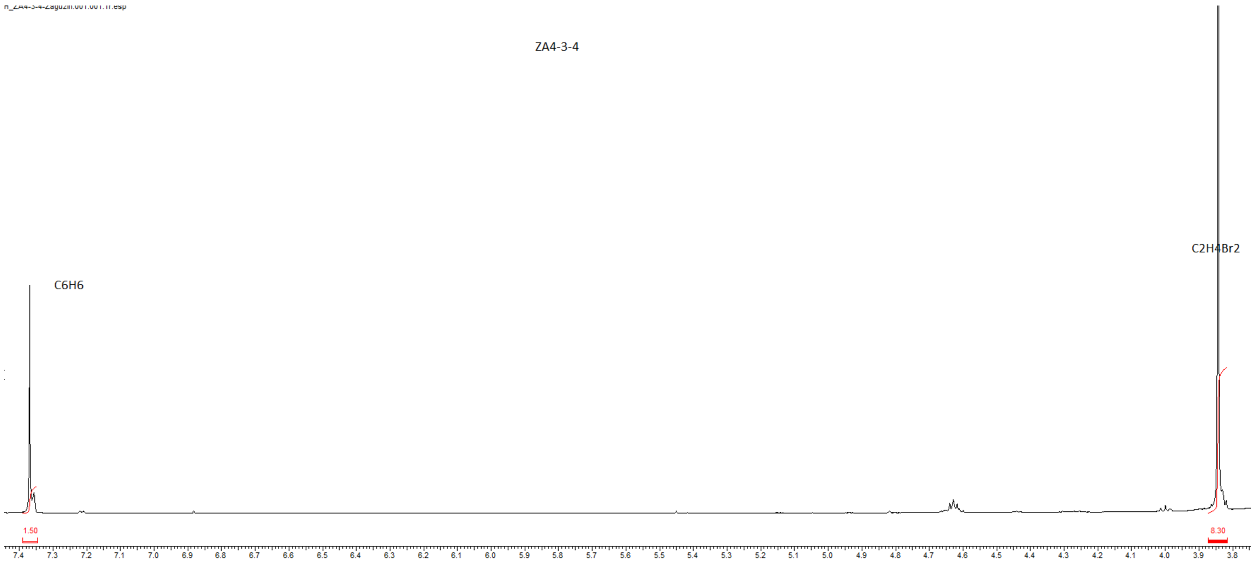

Figure S10. ^{1}H NMR spectrum, sorption experiment with **1**, benzene:dibromoethane

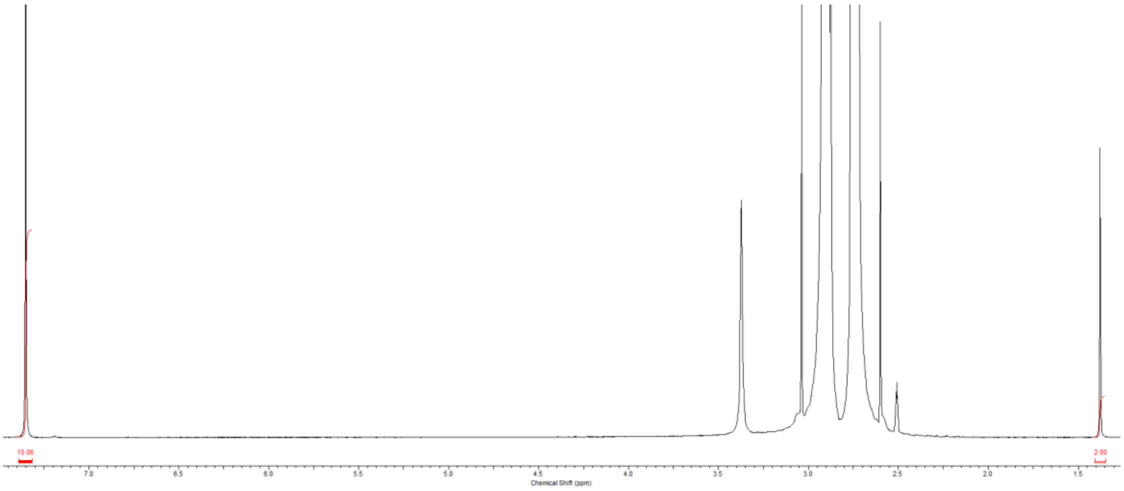

Figure S11. ^{1}H NMR spectrum, sorption experiment with **2**, benzene: cyclohexane

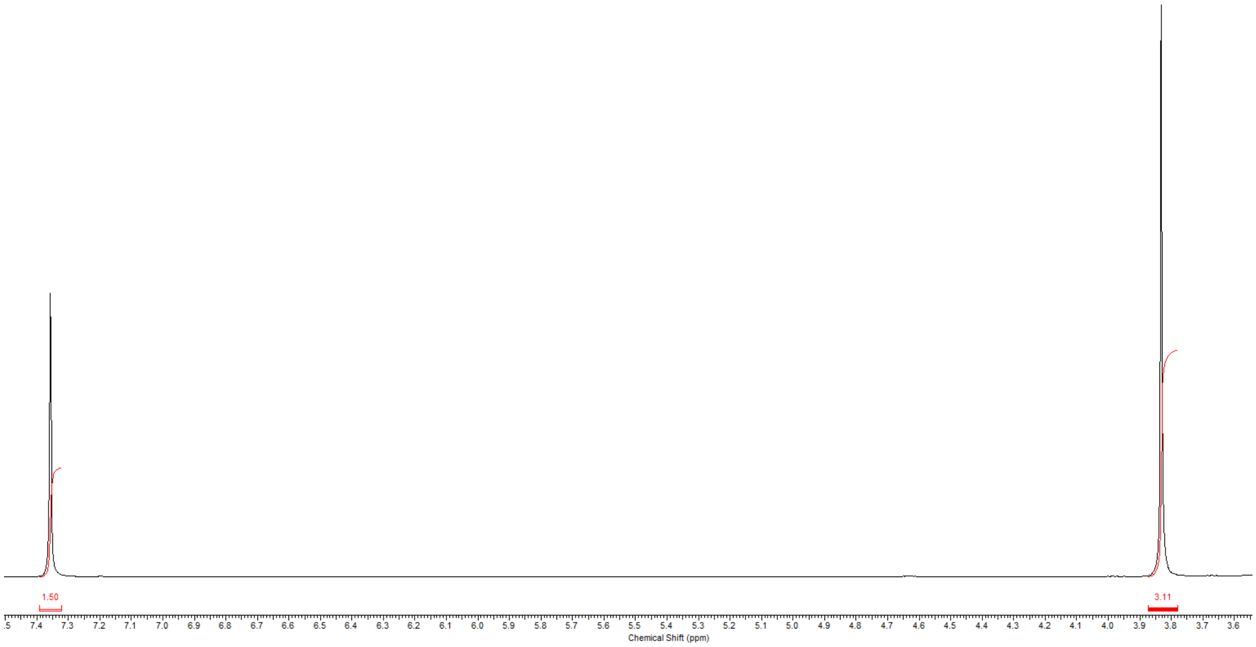

Figure S12. ^{1}H NMR spectrum, sorption experiment with **2**, benzene:dibromoethane

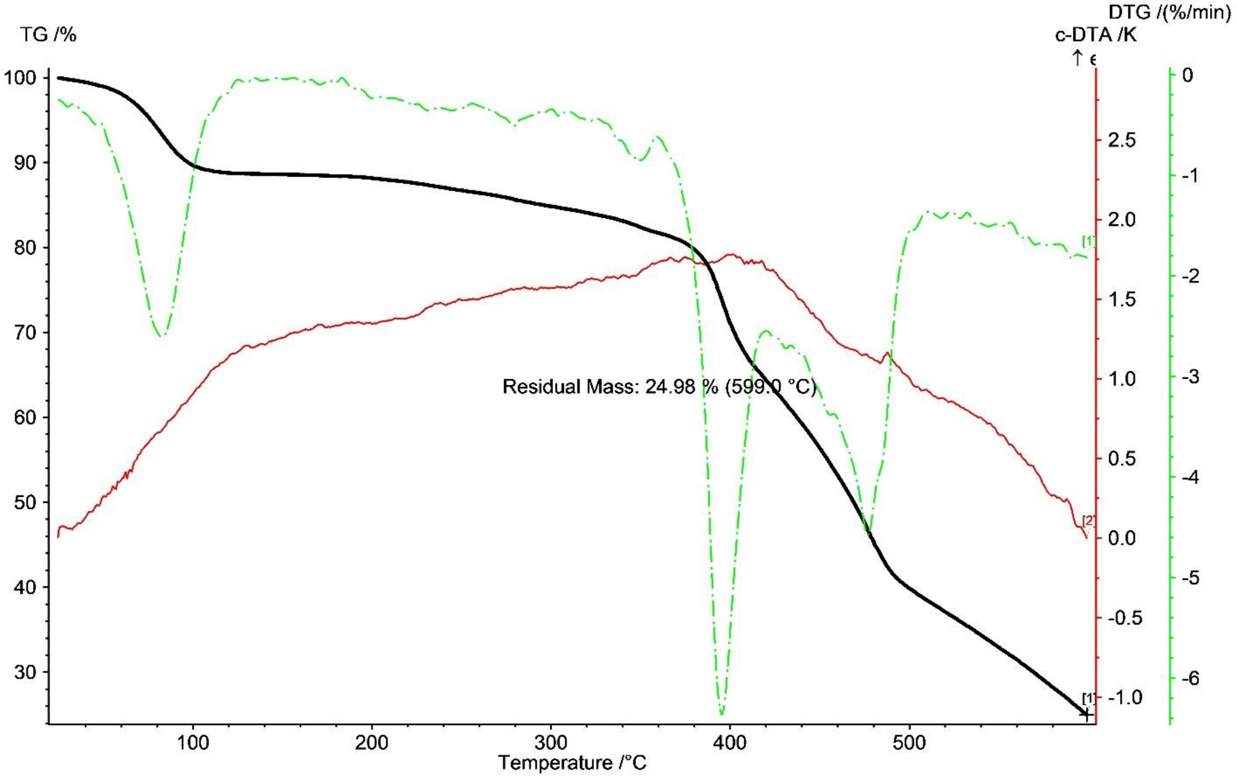

Figure S13. TGA data for as-synthesized **1**

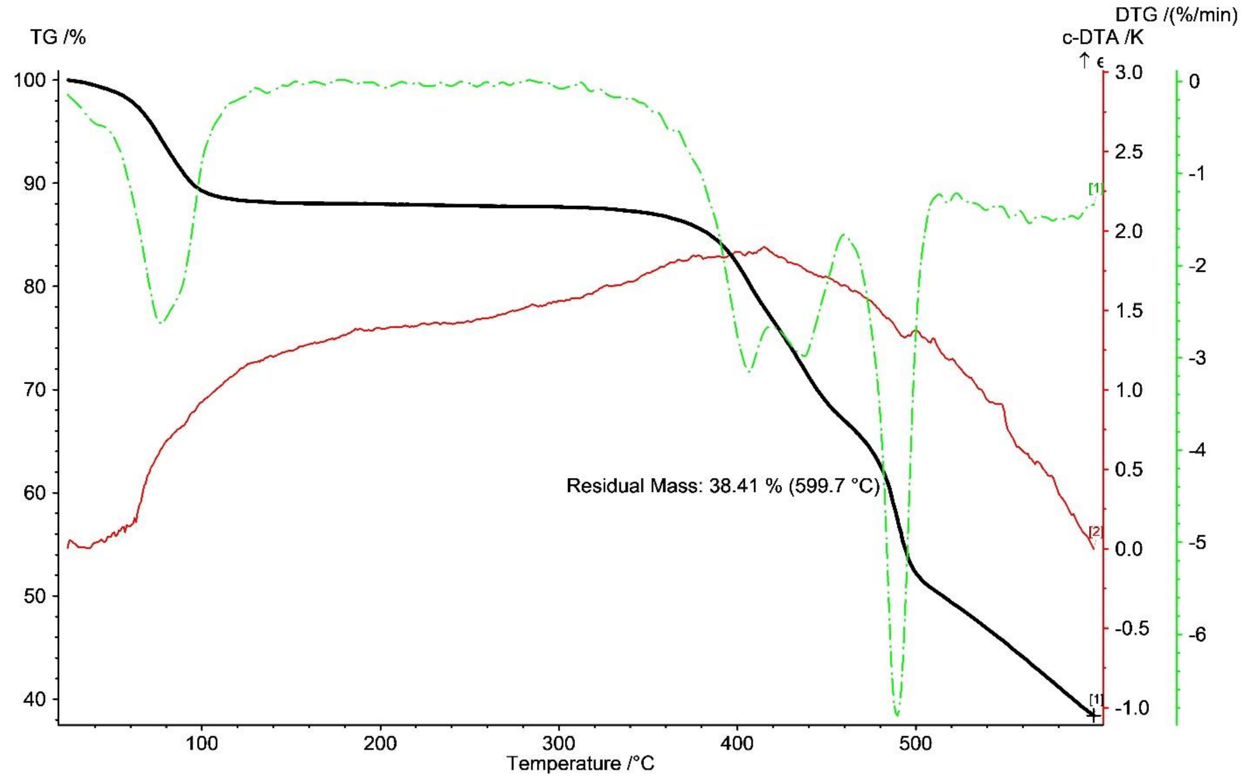

Figure S14. TGA data for as-synthesized **2**
